# Racial and Ethnic Disparities in Fertility Awareness Among Reproductive-Aged Women

**DOI:** 10.1089/whr.2021.0034

**Published:** 2021-08-19

**Authors:** Dana R. Siegel, Jeanelle Sheeder, Alex J. Polotsky

**Affiliations:** Department of Obstetrics and Gynecology, University of Colorado Anschutz Medical Campus, Aurora, Colorado, USA.

**Keywords:** disparities, infertility, knowledge

## Abstract

***Background:*** Despite the rising prevalence of infertility, studies have indicated that in the United States fertility awareness remains low. No published study to date, however, has investigated the impact of any racial or ethnic disparities in fertility awareness.

***Materials and Methods:*** We conducted a cross-sectional survey of people self-identifying as female, aged 18–45 years, *via* Amazon Mechanical Turk in August 2020. The study was approved by the institutional review board at the University of Colorado. The survey consisted of demographic questions and a validated questionnaire, the Fertility and Infertility Treatment Knowledge Score (FIT-KS). Participants were classified as non-Hispanic White (NHW) or “Minority” race/ethnicity.

***Results:*** A total of 476 women completed the survey, 405 of which were included in analysis. Of those, 54.6% self-identified as NHW and 45.4% were in the Minority group. The median FIT-KS was 51.7% (16 items answered correctly). The Minority group scored significantly lower than the NHW participants overall (58.6% vs. 48.3%, *p* < 0.001) and in all three subscales (*p* < 0.05). The Minority group was significantly more likely to underestimate the rate of miscarriage (47.3% vs. 32.6%, *p* = 0.003) and had a lower awareness of risk factors that can impact fertility including smoking (88.7% vs. 71.6%, *p* < 0.001), obesity (90.5% vs. 70.5%, *p* < 0.001), and/or a history of gonorrhea/chlamydia infection (83.7% vs. 64.7%, *p* < 0.001).

***Conclusions:*** Minority women appear to have a lower fertility awareness than their NHW counterparts. Addressing these disparities and improving fertility education in diverse communities may lead to a reduction in clinically significant infertility disparities.

## Introduction

Racial and ethnic disparities exist in many areas of health care, including reproductive medicine. In the United States, these disparities often persist throughout one's reproductive lifetime, from birth to menopause.^1^ The American College of Obstetricians and Gynecologists recognizes the significance of these disparities and reaffirmed in their 2018 committee opinion that raising awareness and reducing racial and ethnic disparities should be a priority for all women's health care providers.^2^

A key example of a racial and ethnic disparity that exists in reproductive medicine is the prevalence, diagnosis, and treatment of infertility.^3^ As women in the United States are continuing to delay childbearing for various reasons, reports have shown that there has been increasing cases of infertility and smaller than desired family sizes.^4,5^ In fact, data from the National Survey of Family Growth highlight that from 2011–2015 to 2015–2017 there has been a rise in the prevalence of impaired fecundity and an increasing percentage of women seeking infertility services.^6,7^ Furthermore, studies have also shown that not only do racial and ethnic minorities including Black and Hispanic women experience infertility significantly more frequently than their Caucasian counterparts, but they are also significantly less likely to receive infertility treatment.^8,9^

Despite the rising prevalence of infertility and subsequent smaller than desired family sizes, fertility awareness defined by the International Glossary on Infertility and Fertility Care as “the understanding of one's reproduction, fecundity, fecundability, and related risk factors” remains low.^10–13^ Using the Fertility and Infertility Treatment Knowledge Score (FIT-KS), Kudesia et al.^11^ highlighted that gaps in fertility knowledge exist in the general reproductive-age U.S. female population, and even among medical trainees. In this seminal study, female medical trainees from two large urban academic medical centers including medical students and obstetric and gynecology residents were found to have substantial gaps in their fertility knowledge.^11^

Notably, meta-analyses have suggested a slight increase in fertility awareness among more educated women and among those who have had difficulties in conceiving; yet to the best of our knowledge, no study to date has investigated the presence of any racial or ethnic disparities in fertility awareness.^10,12,14^

It is imperative to identify and address any meaningful differences in the level of fertility understanding among women from various diverse backgrounds. Increased fertility understanding is likely to help women to make informed decisions regarding planning a desired pregnancy delay, preventing pregnancy when not desired, or actively trying to become pregnant. The overriding objective of our study is to identify any patterns in fertility knowledge gaps among a diverse patient population. Enhanced knowledge of any relevant disparities will serve to enrich individualized reproductive education and counseling regarding fertility-based planning.

## Materials and Methods

### Study design

This was a cross-sectional study using a previously validated questionnaire, the FIT-KS. The study instrument was uploaded to Qualtrics Survey Software^XM^ (Provo, UT) and disseminated to participants living in the United States *via* the Web-based platform Amazon Mechanical Turk (MTurk) from August 6 to 9, 2020. MTurk is an online crowdsourcing forum where requesters can publish and request the completion of various human intelligence tasks, including surveys, to willing participants in the general population. The survey was made public only to registered MTurk users living in the United States who self-identified as female. After completion of the survey in its entirety, the participants were compensated with $1.00. The study was approved by the Colorado Multiple Institutional Review Board.

### Study instrument

The survey was made available in both English and Spanish and consisted of 29 questions surrounding topics related to fertility and infertility risk factors as well as 10 demographic questions. The survey was translated into Spanish by a certified Medical Spanish translator.

The questions were separated into three sections, the first of which covered topics related to natural fertility such as the average rate of fecundability in a given menstrual cycle, the average rate of spontaneous abortion, the fertility window in a given menstrual cycle, and male versus female infertility and contribution to fertilization, among others (20 items). The second group of questions explored topics related to infertility risk factors in a “true” or “false” format including factors, such as tobacco use, obesity, malnutrition, alcohol consumption, caffeine intake, history of sexually transmitted infections, history of pregnancy termination, history of contraception use, and use of certain sexual lubricants (1 item). Finally, the last section explored infertility treatment options as well as their corresponding success rates and cost using the most recent Society for Reproductive Technology data (8 items). The specific questions are available in (Supplementary Appendix SA1).

The questionnaire was designed and published in 2017 by Kudesia et al.^11^ after it underwent extensive review by experts in the field and face validation by the general population living in the United States. First, the questions were ensured to be of appropriate complexity and accuracy by a panel of 15 reproductive endocrinologists. Next, the survey was distributed to 10 participants unaffiliated with the medical field to ensure clarity and was analyzed for item difficulty, instrument reliability, internal consistency, item consistency, and item discriminability. Finally, the survey underwent both discriminative and convergent validity by correlating the total test scores with the level of the participant's gynecologic training as well as by comparing responses to a previously validated survey with several overlapping questions published by Lampic et al.^15^

### Demographic data

In addition, demographic information including race, ethnicity, preferred language, marital status, gravidity, parity, insurance type, and education level was also collected. The classification of race and ethnicity was selected by the participant, and the various options were defined by the investigator, using a similar format to that seen on the U.S. Census Bureau including White, Black or African American, Asian, American Indian, Alaska Native, Native American or Other Pacific Islander, Hispanic or Latinx, or not Hispanic or Latinx. The participant was also given the opportunity to self-describe their racial background if it was not included in the available options. Due to the number of subjects self-identifying in each racial and ethnic minority group, all participants who identified as a race other than non-Hispanic White (NHW) were combined into the Minority group. However, Supplementary Table S1 provides additional information when the results are divided between the groups NHW, non-Hispanic Black (NHB), Hispanic or Latinx (H) if the participant selected any race and Hispanic ethnicity, and non-Hispanic Other (NHO) for those who self-identified as non-Hispanic and a racial group other than White or Black/African American.

Additional inclusion criteria included women between the ages of 18 and 45 years and able to read and write in English or Spanish. The age range of 18–45 years was chosen as this is the age-range that the Centers for Disease Control and Prevention defines as Women of reproductive age. Additionally, the lower limit of 18 years old was also used as according to the latest reports from the Guttmacher Institute, there are still several states in the United States that require parental consent or notification for certain reproductive health services in this population including abortions and contraception.^16^ As some of these topics are explored in the questionnaire, the decision was made to exclude women younger than 18 years to avoid any potential confounding variable such as accessibility.

### Statistical analysis

Responses to items from the FIT-KS were scored as correct or incorrect. These responses were summed, and a percent correct variable was calculated. We computed descriptive statistics including tests of normality for continuous variables. To compare responses of Minority participants with NHW respondents, we used Student's *t*-tests or nonparametric equivalents for continuous variables and chi-squared or Fisher's exact test (for variables with cell sizes <5) for categorical or dichotomous variables. Variables were significant (*p* < 0.1) in bivariate comparisons, and the Minority group was entered into a linear regression model to predict percent correct on the FIT-KS scale. IBM^®^ SPSS Version 27 was used for all analyses.

## Results

### Demographics

The survey was published on the MTurk site, and 476 women completed the survey. Of these, 85% were included in the final analysis (405). The main reason for not being included in the analysis was not meeting the age inclusion criteria (57 respondents were aged 46 years or older and 1 participant aged 17 years or younger). Additionally, 13 participants did not answer the question about their age and were therefore excluded from analysis.

The remaining responses included in analysis were from a diverse group of reproductive-aged women who were predominantly English-speaking, had a college degree or higher, and were privately insured (Table 1). The majority of participants (221, 54.6%) identified themselves as NHW, the reference group. The other 184 respondents (45.4%) self-identified as a race and ethnicity other than NHW, which was categorized as the Minority group. The most common Minority groups included Hispanic White (54, 13.3%) and non-Hispanic Asian (48, 11.9%). Other groups included Hispanic American Indian (30, 7.4%), NHB (25, 6.2%), Hispanic Asian (15, 3.7%), Hispanic Black (8, 2.0%), Hispanic Other (3, 0.7%), and NHO (1, 0.2%) (Table 1).

**Table 1.** Demographics of Study Participants

Characteristic	All responses (N = 405)	NHW (n = 221, 54.6%)	Minority^a^ (n = 184, 45.4%)	p
Age (years)				
18–25	89 (22.0)	34 (15.4)	55 (29.9)	0.008^*^
26–30	95 (23.5)	55 (24.9)	40 (21.7)	
31–35	91 (22.5)	50 (22.6)	41 (22.3)	
36–40	79 (19.5)	49 (22.2)	30 (16.3)	
41–45	51 (12.6)	33 (14.9)	18 (9.8)	
Preferred language				
English	385 (96.0)	212 (97.2)	173 (94.5)	0.33
Spanish	4 (1.0)	1 (0.5)	3 (1.6)	
Other	12 (3.0)	5 (2.3)	7 (3.8)	
Education				
High school or less	22 (5.4)	16 (7.2)	6 (3.3)	0.001^*^
Some college	58 (14.3)	39 (17.6)	19 (10.3)	
College degree	205 (50.6)	117 (52.9)	88 (47.8)	
Master's degree or higher	120 (29.6)	49 (22.2)	71 (38.6)	
Insurance				
Medicaid or Medicare	104 (25.7)	52 (23.5)	52 (28.3)	0.38
Private insurance	251 (62.0)	145 (65.6)	106 (57.6)	
None	47 (11.6)	23 (10.4)	24 (13.0)	
Other	3 (0.7)	1 (0.5)	2 (1.1)	
Desire pregnancy in the next year?				
Yes	140 (34.7)	46 (20.8)	94 (51.4)	<0.001^*^
No	214 (53.0)	149 (67.4)	65 (35.5)	
Undecided	50 (12.4)	26 (11.8)	24 (13.1)	
Gravidity				
0	147 (36.8)	91 (41.6)	56 (31.1)	0.86
1	95 (23.8)	41 (18.7)	54 (30.0)	
2	86 (21.6)	42 (19.2)	44 (24.4)	
3	35 (8.8)	21 (9.6)	14 (7.8)	
4	14 (3.5)	8 (3.7)	6 (3.3)	
≥5	22 (5.5)	16 (7.3)	6 (3.3)	
Parity				
0	162 (40.3)	100 (45.7)	62 (33.9)	0.004^*^
1	118 (29.4)	55 (25.1)	63 (34.4)	
2	83 (20.6)	35 (16.0)	48 (26.2)	
3	23 (5.7)	16 (7.3)	7 (3.8)	
4	9 (2.2)	8 (3.7)	1 (0.5)	
≥5	7 (1.7)	5 (2.2)	2 (1.0)	

^a^ Minority group includes Hispanic Asian, Hispanic American Indian, Hispanic Black, Hispanic Other, Hispanic White, non-Hispanic Asian, non-Hispanic Black, and non-Hispanic Other.

^*^ *p* < 0.05.

NHW, non-Hispanic White.

While there was a fairly even distribution of responses from women of all age categories in both groups, the Minority group was significantly more likely to be <26 years old (29.9% vs. 15.4%, *p* ≤ 0.001). Additionally, the Minority group had a significantly higher number of participants with a master's degree or higher (38.6% vs. 22.2%, *p* < 0.001), but they were significantly less likely to have private insurance (57.6% vs. 65.6%). There was no significant difference between the two groups in terms of language preference (*p* = 0.33). Most participants in both groups spoke English as a preferred language (385, 96.0%, *p* = 0.33), whereas only 4 total participants (1.0%) preferred to take the questionnaire in Spanish. Other preferred languages included Tamil (3), Korean (1), Hindi (1), Portuguese (5), French (1), and Italian (1).

While there was no difference in gravidity overall between the two groups (*p* = 0.086), Minority participants were less likely to be nulligravid (31.1% vs. 41.6%, *p* = 0.031) and nulliparous (33.9% vs. 45.7%, *p* = 0.016). The Minority group was also more likely to desire pregnancy in the next year (20.8% vs. 51.4%, *p* < 0.001) (Table 1).

### Fertility knowledge

Among all respondents, of a maximum possible score of 29, the median score was 51.7% (16 answered correctly), with a range from 24.1% to 86.2% (7–25 answered correctly). Overall, there was a significant difference in the median scores between the NHW and Minority groups, with the Minority group scoring significantly lower than the NHW group (58.6% [24.1%–86.2%] vs. 48.3% [24.1%–79.3%], *p* < 0.001). In logistic regression analysis, having private insurance (adjusted odds ratio [aOR] = 0.159, 95% confidence interval [CI: 0.017 to 0.062]) and nulliparity (*β* = 0.197, 95% CI [0.026 to 0.071]) was positively associated with percent correct, and desiring pregnancy was negatively associated (*β* = −0.184, 95% CI [−0.072 to −0.023]). The Minority group was an independent predictor of percent correct (*β* = −0.21, 95% CI [−0.08 to −0.03]).

**FIG. 1.**
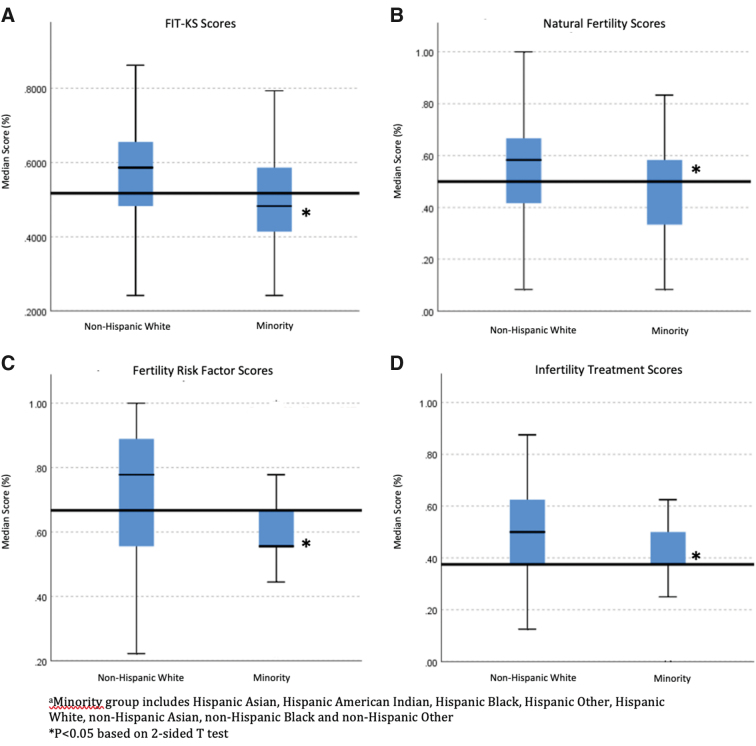
FIT-KS median scores among a diverse group of reproductive-aged females. The Minority group scored significantly lower overall **(A)** as well as on the different categories of questions including those related to natural fertility **(B)**, fertility risk factors **(C)**, and infertility treatment **(D)**. FIT-KS, Fertility and Infertility Treatment Knowledge Score.

When looking at the different categories of questions including those related to natural fertility (questions 1–12), lifestyle risk factors impacting fertility (question 13), and infertility treatment options (questions 14–29), the Minority group scored significantly lower in all three categories (58.3% correct vs. 50%, *p* < 0.001; 77.78% correct vs. 56%, *p* < 0.001; 50% correct vs. 37.5%, *p* < 0.001) (Fig. 1). These differences persisted across the categories of questions when the Minority group was divided into NHB, Hispanic, and NHO with each group scoring lower than the NHW group overall and in each set of questions (Supplementary Table S1).

In regard to natural fertility knowledge, on average, women from both groups tended to underestimate the rate of miscarriage in a reproductive-aged woman (39.3% correct overall) and overestimate the age that fertility declines most precipitously (36.9% correct overall). However, the Minority group was even more likely to overestimate and answer these questions incorrectly (47.3% correct vs. 32.6%, *p* = 0.003; 44.3% correct vs. 27.9%, *p* = 0.001). Additionally, while the majority of both groups were aware that both a man and woman can contribute to a couple's infertility, the Minority group was significantly less likely to answer this question correctly (97.7% vs. 92.8%, *p* = 0.018).

In terms of lifestyle factors that can impact fertility, the Minority group scored significantly lower on questions related to risk factors that can decrease a woman's chance of fertility, including smoking (88.7% correct vs. 71.6%, *p* < 0.001), obesity (90.5% correct vs. 70.5%, *p* < 0.001), and/or a history of gonorrhea/chlamydia infection (83.7% correct vs. 64.7%, *p* < 0.001). Approximately half of participants overall believed that safe pregnancy termination can negatively impact a woman's fertility, yet the Minority group was significantly more likely to incorrectly answer that this statement is true (63.2% correct vs. 37%, *p* < 0.001).

With regard to infertility treatment options, respondents were generally aware of the artificial reproductive technology (ART) options and definitions; however, the Minority group was less familiar with intrauterine insemination (70.6% correct vs. 49.5%, *p* < 0.001) and egg cryopreservation (70.6% correct vs. 44%, *p* < 0.001). In general, respondents tended to overestimate the success of ART and underestimate the chance of twins. Furthermore, the NHW group was significantly more likely to overestimate the average price of one *in vitro* fertilization (IVF) cycle in the United States (39.4% correct vs. 58.2%, *p* < 0.001).

## Discussion

Health care disparities have been recognized as one of the greatest challenges that we face in the 21st century and addressing these differences has been a priority of the National Institutes of Health for many years. However, racial and ethnic disparities continue to persist in many areas of reproductive medicine, including the prevalence of infertility, the access to infertility services, and the success of infertility treatment among racial and ethnic minorities.^8,9,17–19^ Using the validated FIT-KS survey published in 2017 by Kudesia et al.,^11^ the results from this cross-sectional study are a novel finding that racial and ethnic disparities also exist in fertility awareness, or the understanding of one's reproductive potential and related risk factors. It is suggested that these differences in knowledge may contribute to the above disparities seen in clinical practice.

Overall, in the several years since the initial publication of the FIT-KS instrument, fertility-related knowledge among reproductive-aged women living in the United States has remained unchanged. Of a maximum score of 29, participants from the study of Kudesia et al.^11^ in the general population had a median score of 55.9% correct, with medical trainees scoring slightly higher at 64.9%. However, the present study highlights that fertility knowledge has actually decreased to a median score of 51.7% correct. While several organizations have been developed to promote fertility awareness, including the Fertility Appreciation Collaborative to Teach the Science, this persistence of low fertility awareness overall emphasizes the need for further national implementation of educational services and continued counseling.

Importantly, the Minority group in this study, defined as a race and ethnicity other than NHW, scored significantly lower in all areas of fertility awareness, including questions related to natural fertility, infertility risk factors, and infertility treatment. Additionally, when separating the Minority group into those who self-identified as NHB, Hispanic, and non-Hispanic along with a racial group other than White or Black, these differences persisted. While other studies have shown that fertility awareness is higher among more educated women, the results from our study highlight that racial and ethnic disparities in fertility awareness persisted regardless of educational level as the Minority group was significantly more likely to have a master's degree or higher. These differences also persisted despite the Minority group being significantly more likely to desire pregnancy in the next year, which other studies have suggested may improve fertility awareness.^12^

While reproductive-aged women overall continue to overestimate the fecundability of women at age 30 years or older, the Minority group was significantly more likely to overestimate the age that a woman's ability to get pregnant declines most precipitously. This becomes important for future fertility planning as women continue to postpone childbearing for various reasons.^20^ However, it may be even more significant for Minority women as Black, Asian, and Hispanic women endure infertility for a longer period of time, on average, than White women before seeking infertility services.^9,21,22^ As Minority women are less likely to pursue a formal infertility workup, it is important to understand how to best take advantage of one's menstrual cycle to increase the chance of pregnancy. However, this study highlights the significant misunderstanding that many women, especially Minority women, face when considering the optimal timing for intercourse as only 38% of the Minority group answered this question correctly.

Furthermore, the Minority population was significantly less likely to correctly identify that a history of gonorrhea or chlamydia infection may impact one's future fertility. This knowledge gap may contribute to the clinical differences seen in infertility diagnoses as a significantly greater proportion of Black and Hispanic women have been shown to have tubal factor infertility compared with White women, which is a preventable cause of infertility.^9^ Additionally, the Minority group's lower awareness of an elevated body mass index contributing to infertility may also translate clinically to the lower success rate, defined as live birth, of obese Black and Hispanic women undergoing IVF and the higher doses of gonadotropins required by Black women undergoing nondonor IVF.^17,19^

Finally, our study also highlights that NHW women are more familiar with the various infertility treatment options that exist. Although many complex sociodemographic and cultural barriers play a role in the equitable access to infertility services, an additional reason why NHW women may be more likely to seek infertility care suggested by our findings is that this group of women also tends to overestimate the success rates of artificial reproductive technology (ART) and are less familiar with the average cost of an IVF cycle than the Minority women. NHW women are more likely to be privately insured than the Minority women, and findings from our study suggest that private insurance may portend a higher fertility awareness.^23^ However, a previous study by Jain and Hornstein highlighted that insurance status may not explain the disparities in access to infertility services as states such as Massachusetts, with mandated insurance coverage for infertility services, the predominant client seeking these services continues to be the NHW population.^24^

While this was a novel study highlighting racial and ethnic disparities in fertility-related knowledge, there certainly were several limitations. To start, the data collection platform MTurk used to obtain the survey responses may recruit participants who are not representative of the U.S. population and may therefore hinder the generalizability and external validity of such findings. To complete a survey published on MTurk, one must have reliable access to internet and a functional computer, tablet, or smartphone. MTurk participants, therefore, on average tend to be more highly educated and younger than the national population.^25^ While MTurk participants are compensated and rated by their thorough completion of published tasks, the workers are completing such tasks in an unsupervised environment with little verification surrounding the accuracy of such responses. Nonetheless, many studies have shown MTurk to be a trustworthy and reliable source of information that may even be superior to that of other samples and has now become the largest online crowdsourcing platform in the world.^26^ Furthermore, while the FIT-KS questionnaire was first reviewed by a panel of reproductive endocrinologists for appropriate depth and then administered to laypeople for face validation in the original study, the questions are designed to be quite difficult and therefore may not accurately reflect a clinically relevant fertility understanding among the general population. Finally, although in our supplementary analysis we found that the differences in fertility awareness among the Minority group persisted when divided into NHB, Hispanic, and NHO, the study was not adequately powered to address any specific differences among the individual racial or ethnic Minority groups. Despite these limitations, we believe that the findings from this study highlight the need for culturally competent and individualized counseling in reproductive and fertility-based education. More research is needed to investigate possible nuances in the various racial and ethnic Minority groups and to determine the most effective interventions to improve fertility awareness among an ever-increasing diverse population.

## Conclusions

Fertility awareness overall remains quite low among reproductive-aged women in the United States, and no improvements in these knowledge gaps have been made over the past several of years. Importantly, these knowledge gaps are even more significant among racial and ethnic Minority women. Addressing these disparities and improving fertility education in underserved communities may lead to a reduction in clinically significant infertility disparities. Not only can improved fertility awareness help those who are actively trying to become pregnant achieve their goal of parenthood, but it may also provide information to those avoiding or delaying pregnancy as more women are relying on natural family planning to prevent an unwanted pregnancy. Further work is needed regarding the development and implementation of culturally sensitive and individualized reproductive education and counseling regarding fertility-based planning.

## Supplementary Material

Supplemental data

Supplemental data

## Supplementary Material

Supplementary Table S1

Supplementary Appendix SAP1

## Abbreviations Used

FIT-KS: Fertility and Infertility Treatment Knowledge Score
IVF: *in vitro* fertilization
MTurk: Mechanical Turk
NHB: non-Hispanic Black
NHO: non-Hispanic Other
NHW: non-Hispanic White
