# Supplementary material for: Racial and Ethnic Disparities in Fertility Awareness Among Reproductive-Aged Women
Source: Womens Health Rep (New Rochelle). 2021 Aug 19;2(1):347–54. doi: 10.1089/whr.2021.0034 (PMC8409232; doi:10.1089/whr.2021.0034)
Supplement: Supplemental data [file Supp_AppS1.docx]

**The following questions relate to natural fertility: // *Las siguientes preguntas se relacionan con la fertilidad natural:***

1. At what age are women most fertile? // *¿A qué edad son las mujeres más fértiles?*

□ 12-19

□ 20-29*

□ 30-39

□ 40-49

2. Over which age range does a woman’s ability to get pregnant decline most precipitously? // *¿Sobre qué rango de edad la capacidad de una mujer para quedar embarazada disminuye más precipitadamente?*

□ 25-29

□ 30-34

□ 35-39*

□ 40-45

3. Over the course of 1 month, what is the percent chance that a 30 yo woman who is trying to get pregnant will get pregnant? // *En el transcurso de 1 mes, ¿cuál es el porcentaje de probabilidad de que una mujer de 30 años que está tratando de quedar embarazada quede embarazada?*

□ 10%

□ 20%*

□ 30%

□ 40%

4. Over the course of 1 month, what is the percent chance that a 40yo woman who is trying to get pregnant will get pregnant? // *En el transcurso de 1 mes, ¿cuál es el porcentaje de probabilidad de que una mujer de 40 años que está tratando de quedar embarazada quede embarazada?*

□ ≤5%*

□ 6-10%

□ 11-15%

□ 16-20%

5. On average, for a woman in her peak reproductive years, what is the percent chance that a pregnancy (recognized or unrecognized) will end in a miscarriage? // *En promedio, para una mujer en sus mejores años reproductivos, ¿cuál es el porcentaje de probabilidad de que un embarazo (reconocido o no reconocido) termine en un aborto espontáneo?*

□ ≤5%

□ 6-15%

□ 16-25%*

□ 26-35%

6. A woman and a man can both contribute to a couple's infertility: // *Una mujer y un hombre pueden contribuir a la infertilidad de una pareja:*

□ True* // Cierto*

□ False // Falso

7. A man’s age is a factor that affects a couple’s fertility: // *La edad de un hombre es un factor que afecta la fertilidad de una pareja:*

□ True* // Cierto*

□ False // Falso

8. Having less than 9 periods in a year can be normal for some women and doesn’t require any further evaluation: // *Tener menos de 9 períodos en un año puede ser normal para algunas mujeres y no requiere ninguna evaluación adicional:*

□ True // Cierto

□ False* // Falso*

9. What is the average survival time of normal sperm in the female reproductive tract? // *¿Cuál es el tiempo de supervivencia promedio de los espermatozoides normales en el tracto reproductivo femenino?*

□ 12-24 hours // 12-24 horas

□ 24-48 hours // 24-48 hours

□ 3-5 days* // 3-5 días*

□ 6-9 days // 6-9 días

10. When is the optimal time to have sexual intercourse in order to get pregnant? // *¿Cuándo es el momento óptimo para tener relaciones sexuales para quedar embarazada?*

□ Right before the period starts // *Justo antes de que comience el período*

□ First few days of the period // *Primeros días del período*

□ About halfway through the cycle* // Aproximadamente a la mitad del ciclo *

□ It doesn’t matter // *No importa*

11. Where does fertilization most commonly occur? // *¿Dónde ocurre la fertilización más comúnmente?*

□ In the uterus // *En el útero*

□ Inside the ovaries // *Dentro de los ovarios*

□ On the surface of the ovaries // *En la superficie de los ovarios*

□ In the Fallopian tubes* // *En las trompas de Falopio*

12. How many eggs are typically released per cycle? // *¿Cuántos huevos se liberan típicamente por ciclo?*

□ 1*

□ 2

□ 3

□ 4

**The following are likely to decrease a woman’s chance of fertility: // *Es probable que lo siguiente disminuya las posibilidades de fertilidad de una mujer:***

True/*Cierto* False/*Falso*

13. Smoking // *Fumando*………………………………………………. □* □

14. Occasional caffeine intake // *Ingesta ocasional de cafeína* ……….. □ □*

15. Moderate alcohol consumption // *Consumo moderado de alcohol*… □ □*

16. Safe pregnancy termination // *Interrupción segura del embarazo*… □ □*

17. Obesity // *Obesidad*……………………………………………....... □* □

18. Gonorrhea/Chlamydia infection // *Infección por gonorrea/clamidia*. □* □

19. Prior use of contraceptive pills //

*Uso previo de píldoras anticonceptivas…………………………………….* □ □*

20. Being underweight due to frequent exercise

or limited caloric intake // *Tener bajo peso debido al*

*ejercicio frecuente o ingesta calórica limitada*……………………….… □* □

21. Using certain types of sexual lubricants //

*Usar ciertos tipos de lubricantes sexuales*…………………..……….….. □* □

**The remainder of questions relate to infertility treatment: // *El resto de las preguntas se relacionan con el tratamiento de infertilidad:***

22. In vitro fertilization (IVF) refers to a treatment in which: // *La fertilización in vitro (FIV) se refiere a un tratamiento en el que:*

□ A thin catheter is used to deposit a man’s sperm past the cervix directly into the uterus // *Se usa*

*un catéter delgado para depositar el esperma de un hombre más allá del cuello uterino directamente*

*en el útero*

□ A man’s sperm and a woman’s egg are combined inside a laboratory and the resulting embryo is transferred into the uterus* // *El esperma de un hombre y el óvulo de una mujer se combinan dentro de un laboratorio y el embrión resultante se transfiere al útero**

□ Sperm are deposited directly into the vagina (“turkey baster”) // *Los espermatozoides se depositan directamente en la vagina ("pavo baster")*

□ Surgery is performed to harvest sperm from the man // *La cirugía se realiza para recolectar esperma del hombre.*

**The following 3 questions refer to the most recent national statistics published by the Centers for Disease Control & Prevention and the Society for Assisted Reproductive Technology: //
*Las siguientes 3 preguntas se refieren a las estadísticas nacionales más recientes publicadas por los Centros para el Control y la Prevención de Enfermedades y la Sociedad de Tecnología de Reproducción Asistida:***

23. For a woman under 35 years old, undergoing IVF with her own eggs, what is the pregnancy rate per cycle? // *¿Para una mujer menor de 35 anos de edad, que se someta a FIV con sus propias ovulos, que es el tasa de embarazo por ciclo?*

□ ≤5%

□ 6-20%

□ 21-40%

□ 41-60%*

□ ≥ 60%

24. For a woman over 44 years old, undergoing IVF with her own eggs, what is the pregnancy rate per cycle? // *¿Para una mujer mayor de 44 anos que se somete a FIV con sus propias ovulos, cual es la tasa de embarazo por ciclo?*

□ ≤5%*

□ 6-20%

□ 21-40%

□ 41-60%

□ ≥ 60%

25. In women who are undergoing IVF, what is the percent of pregnancies that result in twins? // *En las mujeres que se someten a FIV, ¿cuál es el porcentaje de embarazos que resultan en gemelos?*

□ ≤5%

□ 6-20%

□ 21-35%*

□ 36-45%

26. What is the average cost of an IVF cycle in the USA? // *¿Cuál es el costo promedio de un ciclo de FIV en los Estados Unidos?*

□ $5,000

□ $12,000*

□ $20,000

□ $50,000

27. Intrauterine insemination (IUI) refers to a treatment in which: // *La inseminación intrauterina (IUI) se refiere a un tratamiento en el que:*

□ A thin catheter is used to deposit a man’s sperm past the cervix directly into the uterus* // *Se usa*

*un catéter delgado para depositar el esperma de un hombre más allá del cuello uterino directamente*

*en el útero **

□ A man’s sperm and a woman’s egg are combined inside a laboratory and the resulting embryo is transferred into the uterus // *El esperma de un hombre y el óvulo de una mujer se combinan dentro de un laboratorio y el embrión resultante se transfiere al útero.*

□ Sperm are deposited directly into the vagina (“turkey baster”) // *Los espermatozoides se depositan directamente en la vagina ("pavo baster")*

□ Surgery is performed to harvest sperm from the man // *La cirugía se realiza para recolectar*

*esperma del hombre.*

28. Egg cryopreservation (freezing) refers to an infertility treatment in which: // *La criopreservación del huevo (congelación) se refiere a un tratamiento de infertilidad en el que*

□ A single sperm is injected into an egg to preserve the egg’s integrity // *Se inyecta un solo esperma en un huevo para preservar la integridad del huevo*

□ A man’s sperm and woman’s egg are combined inside a laboratory and then frozen // *El esperma de un hombre y el óvulo de una mujer se combinan dentro de un laboratorio y luego se congelan*

□ Strips of ovarian tissue are surgically removed and frozen // *Las tiras de tejido ovárico se extirpan quirúrgicamente y se congelan*

□ Eggs are frozen following ovarian stimulation and egg retrieval* // *Los huevos se congelan después de la estimulación ovárica y la recuperación de los huevos **

29. When using frozen eggs from women less than 37 years old, what is the live birth rate per thawed egg? // *Cuando se usan huevos congelados de mujeres menores de 37 años, ¿cuál es la tasa de nacimientos vivos por huevo descongelado?*

□ ≤10%*

□ 11-15%

□ 16-20%

□ 21-25%

-----------------------------------------------------------------------------------------------------------------------------

30. Age (years) // *Edad (años)*

□ Age 17 or younger

□ 18-25

□ 26-30

□ 31-35

□ 36-40

□ 41-45

□ Age 46 or older

31. What is your marital status? // *¿Cuál es su estado civil?*

□ Single // *Soltera*

□ In a relationship // *En una relación*

□ Married // Casada

□ Divorced or separated //*Divorciada o separada*

□ Widowed // *Viuda*

32. What is your preferred language? // *¿Cuál es su idioma preferido?*

□ English // *Inglés*

□ Spanish // Español

□ Other:_______ // *Otro:* _______

33. What is your race? // *¿Cuál es su raza?*

□ White // *Raza blanca*

□ Black or African-American // Negra o *Afroamericana*

□ Asian // Asiática

□ American Indian // Amerindio

□ Alaska Native // Indígena de Alaska

□ Native Hawaiian and Other Pacific Islander // Nativo de Hawai y otras islas del Pacífico

□ Other:_______ // *Otro:* _______

34. What is your ethnicity? // *¿Cuál es su origen étnico?*

□ Hispanic or Latinx // Hispana/o o Latinx

□ Not Hispanic or Latinx // No Hispana/o o Latinx

35. Education // *Educación*

□ High school or less // *Escuela secundaria o menos*

□ Some college // *alguna educación superior*

□ College degree // *título universitario*

□ Masters degree or higher// *licenciatura o superior*

36. What type of insurance do you have? *¿Qué tipo de seguro tiene?*

□ Medicaid or Medicare // *Medicaid o Medicare*

□ Private Insurance // *Seguro privado*

□ None // *Ninguno*

□ Other: _______ // *Otro:* _______

37. Would you like to become pregnant in the next year? // *¿Le gustaría estar embarazada en el próximo año?*

□ Yes // *Sí*

□ No // *No*

□ Undecided // *Indecisa*

38. How many times have you been pregnant? _______ // *¿Cuántas veces ha estado embarazada?* _______

39. How many children do you have? _______ // *¿Cuántos hijos tiene?* _______
