# Supplementary material for: Racial and Ethnic Disparities in Fertility Awareness Among Reproductive-Aged Women
Source: Womens Health Rep (New Rochelle). 2021 Aug 19;2(1):347–54. doi: 10.1089/whr.2021.0034 (PMC8409232; doi:10.1089/whr.2021.0034)
Supplement: Supplemental data [file Supp_TableS1.docx]

**Supplementary Table 1.** Fertility & Infertility Treatment Knowledge Score (FIT-KS) median scores across racial and ethnic groups

| **Instrument Median Score** | **Non-Hispanic White**  **(n=221; 54.6%)** | **Non-Hispanic Black**  **(n=25; 6.2%)** | **Hispanic**  **(n=111; 27.4%)** | **Non-Hispanic Other^a^**  **(n=48; 11.9%)** | **p value** |
| --- | --- | --- | --- | --- | --- |
|  | % correct  Median (range) | % correct  Median (range) | % correct  Median (range) | % correct  Median (range) |  |
| **Overall** | 58.6 (24-86) | 51.7(31-72) | 48.3 (24-79) | 56.9 (31-76) | <0.001* |
| **Natural Fertility** | 58.3 (8-100) | 41.7 (17-75) | 50 (8-83) | 50 (17-83) | <0.001* |
| **Infertility Risk Factors** | 77.8 (22-100) | 55.6 (22-89) | 55.6 (22-89) | 55.6 (33-100) | <0.001* |
| **Infertility Treatment** | 50.0 (0-88) | 50 (13-75) | 37.5 (0-88) | 50.0 (13-75) | <0.001* |

^a^ Non-Hispanic Other includes those who self-identified as non-Hispanic and a racial group other than White or Black/African American.

* p < 0.05
